# Habitat suitability analysis of *Dendrobium flexicaule* using MaxEnt and InVEST models

**DOI:** 10.3389/fpls.2025.1686507

**Published:** 2025-11-20

**Authors:** Wei Lin, Chenjuan Ye, Xingjia Ming, Junsheng Qi, Xiang Liu, Guochun Fan, Jiaxin Liao, Yongde Wang, Xue Liu

**Affiliations:** 1 Chongqing Key Laboratory of special Chinese Materia medica resources utilization and evaluation, Endangered Medicinal Breeding National Engineering Laboratory, Chongqing Academy of Chinese Materia Medica, Chongqing, China; 2 College of Biology and Food Engineering, Chongqing Three Gorges University, Chongqing, China; 3 Daba mountain Medicinal Animals and Plants of Chengkou Observation and Research Station, Chongqing Academy of Chinese Materia Medica, Chongqing, China; 4 Chongqing Daba Mountain National Nature Reserve Administration Center, Chongqing, China

**Keywords:** *Dendrobium flexicaule* Z. H. Tsi, S. C. Sun & L. G. Xu, suitability, MaxEnt, InVEST, conservation, development

## Abstract

**Introduction:**

*Dendrobium flexicaule*, an orchid endemic to China, is valued in Traditional Chinese Medicine; however, its conservation and sustainable use lack spatial guidance.

**Methods:**

This study employed the MaxEnt model to predict suitable habitats for *D. flexicaule* under varying climatic conditions. Building upon the high-suitability areas identified by MaxEnt, we further utilized the InVEST model to delineate high-quality habitat zones as key areas for conservation and development planning.

**Results:**

Results revealed that *D. flexicaule* is primarily found in Sichuan, Chongqing, Guizhou, Henan, and Hubei. Future scenarios suggest an expansion of suitable habitats, with precipitation, temperature, and slope emerging as the dominant environmental drivers. Priority conservation zones cluster along the southern and the eastern edge of the Qinba Mountains, whereas priority development areas concentrate in the northern foothills of the Qinba Mountains.

**Discussion:**

Our analysis highlighted the critical influence of precipitation and temperature on the species’ distribution, ultimately identifying priority conservation areas. This integrated approach provides a scientific foundation for the sustainable development of *D. flexicaule*.

## 1 Introduction

In China, a total of 74 species and two varieties of *Dendrobium* are recorded, primarily distributed in the Qinling Mountains, the Yangtze River Basin, and adjacent southern regions. These taxa typically occupy areas between 15°30′ and 25°12′ N, gradually extending northward with a concomitant decline in species diversity. Compared with other Dendrobium species, D. flexicaule inhabits notably higher latitudes. Its core distribution is centered in the Qinba Mountains, extending southward along the Hengduan Mountains to Binchuan (Dali, Yunnan, China). The species is a habitat specialist, growing as a lithophyte on north-facing cliff faces (Zhang, 1995; Zhang, 2016). It is among the key original plant sources of the Chinese medicinal material “*D. flexicaule*,” valued not only for its ornamental potential but also for its substantial medicinal properties (Editorial Committee of the Flora of China, Chinese Academy of Sciences, 1999). The stems of *D. flexicaule*, renowned for their cold nature and sweet taste, are said to nourish yin and yang, rejuvenate the liver and kidneys, and are traditionally consumed to promote longevity. Folk medicine further employs these stems to treat infantile convulsions. Chemical analyses have revealed numerous alkaloids, polysaccharides, and 17 free amino acids in the plant. When boiled, it yields a fragrant, sweet broth described as “delicious.” As early as the Shennong Bencao Jing, *D. flexicaule* was classified as a top-grade herb. However, this species is increasingly threatened by habitat specificity, limited distribution, slow growth rates, and excessive long-term harvesting. In 2021, it was officially recognized as a national first-class protected plant in China (http://www.forestry.gov.cn/main/5461/20210908/162515850572900.html) and is also listed as endangered by the IUCN (https://www.iucnredlist.org). Endemic plants are typically confined to narrow geographical areas and are often rare or endangered (Zhu and Sheng, 2019). Their distribution patterns are crucial for floristic regionalization and vegetation zoning, and understanding the characteristics, origin, formation, and evolution of flora (Wu, 1991; Brooks et al., 2006; Lamoreux et al., 2006). As a biodiversity hotspot, the Qinba Mountains' conservation priority is partly determined by its number of endemic species.Research on the distribution of *D. flexicaule*, an endangered and endemic plant in China, not only aids in its protection but also offers theoretical and decision-making support for the Qinba Mountain region's biodiversity conservation strategy. This ensures the protection of endemic species and promotes the sustainable development of biodiversity.

Species distributions emerge from complex interactions among evolutionary processes, human interventions, and a spectrum of environmental parameters—encompassing climate, terrain, soil characteristics, biotic factors, and migration histories. These distributions reflect the evolutionary trajectory, population dynamics, and ecological adaptability of species (Soberon and Peterson, 2005). Among environmental influences, climate variables such as precipitation and temperature crucially shape plant growth, development, and distribution (Bertrand et al., 2011; Lenoir et al., 2008). Current greenhouse gas emissions have led to global warming, an escalating environmental challenge particularly for plant species whose already restricted habitats may contract further, intensifying their risk of endangerment (Ashraf et al., 2016; Chichorro et al., 2019; Fitzpatrick et al., 2008). In response to rising temperatures, certain species migrate to higher latitudes or elevations (Bertrand et al., 2011; Root et al., 2003), although these shifts manifest differently across taxa. Consequently, understanding species-specific climate-change responses is critical to biodiversity conservation efforts. In recent decades, Ecological niche models (ENMs), commonly termed species distribution models (SDMs), have been extensively employed to predict suitable habitats (Raes, 2012). Of these, the MaxEnt model has gained particular prominence due to its robust predictive power and high spatial consistency (Liu et al., 2019; Phillips and Dudík, 2008). Compared with other models, MaxEnt requires limited occurrence data (n ≥ 5), is cost-efficient, easy to use, runs quickly, and generally offers superior results (Liu Ran et al., 2018; Pearson et al., 2007). Researchers have increasingly adopted MaxEnt to map suitable regions for medicinal plants and to analyze how these species respond to climate change, even under conditions of limited data (Li et al., 2020a; Liu et al., 2021).

Amid ongoing global habitat loss (Field and Barros, 2014; Newbold et al., 2015), identification of priority conservation and development areas remains a key strategy for enhancing nature reserve management and achieving biodiversity objectives (Brooks et al., 2006; Ma et al., 2021). The InVEST model, which requires relatively minimal data yet yields high assessment accuracy, has become a popular tool for habitat quality evaluation (Wang et al., 2024). Scholars have employed it at diverse spatial scales—ranging from nature reserves (Gu et al., 2019) and counties (Luan et al., 2023) to urban agglomerations (Wu et al., 2015) and river basins (Xu et al., 2023a). Because land-use patterns greatly impact habitat quality, integrating the InVEST model with land-use analyses can effectively delineate priority conservation and development zones, demonstrating strong operational feasibility.

Against this backdrop, the aims of the present study are threefold: (1) Analyze the distribution of suitable areas for *D. flexicaule* under different climate scenarios and evaluate their temporal dynamics; (2) Identify key factors that affect its distribution; and (3) integrate MaxEnt projections with the InVEST model to define priority conservation and development regions for this species. These findings will not only augment our understanding of how *D. flexicaule* may respond to extreme climate events and other environmental pressures but also provide a foundational reference for future studies on biodiversity, species differentiation, and the broader conservation of this medically and ecologically significant orchid.

## 2 Data and models

### 2.1 Research framework

**Figure 1:**
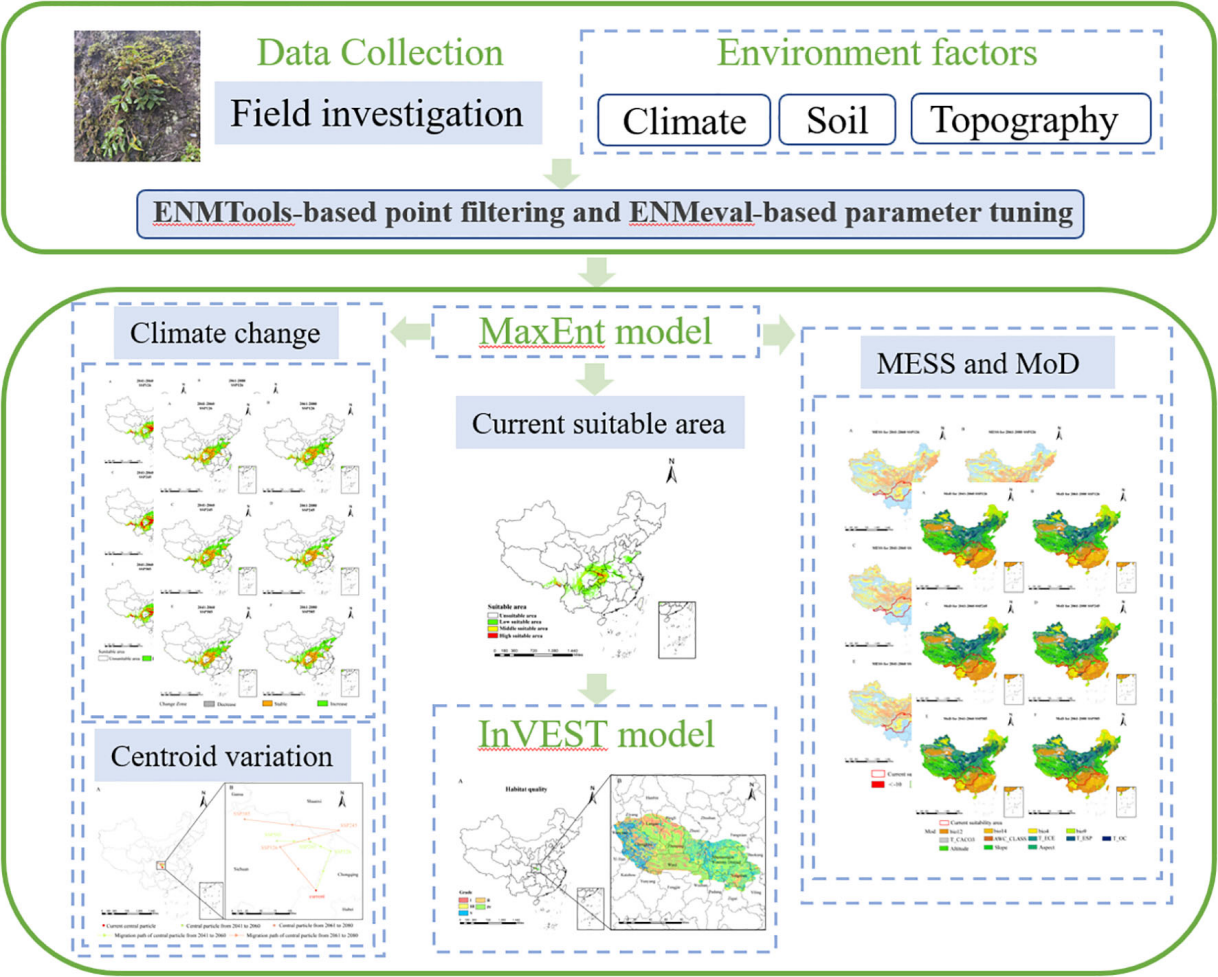
Image summary.

We developed an integrated modeling workflow by coupling species habitat suitability predictions (MaxEnt) with Habitat quality assessment (InVEST). This framework enabled spatially explicit mapping of *D. flexicaule ‘s* potential range under current bioclimatic conditions and future scenarios, explored the priority conservation and development areas of this species (**Figure 1**).

### 2.2 Data processing

#### 2.2.1 Data and distribution point processing of *D. flexicaule* with curved stem

**Figure 2:**
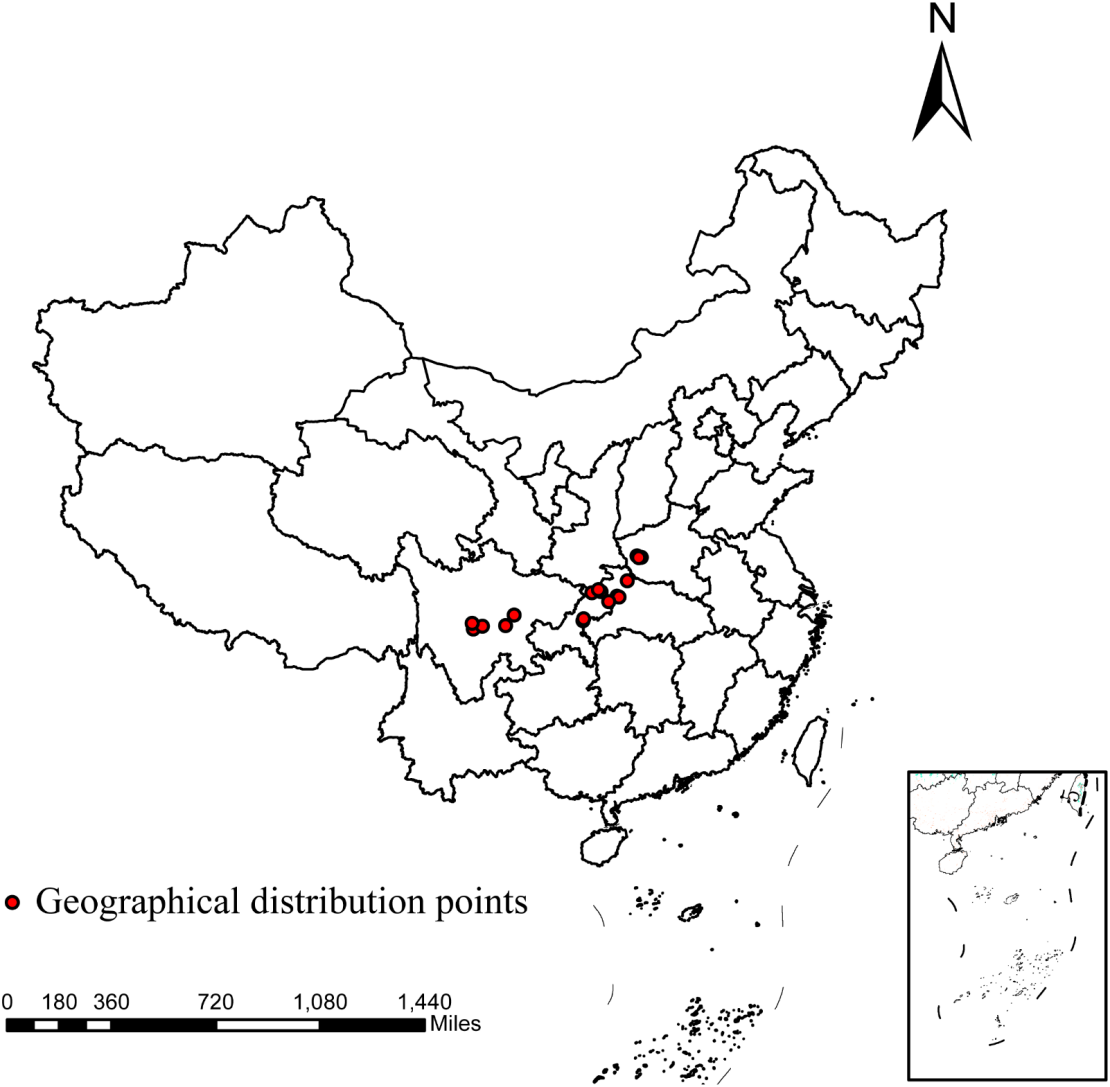
Geographical distribution points of *D.flexicaule.* Map creation using ArcMap 10.8.0 (URL: https://www.arcgis.com/index.html). China Administrative Map from Resource and Environmental Science Data Platform (https://www.resdc.cn/). GS (2024)0650.

The distribution data for *D. flexicaule* were gathered during field surveys by the project team using handheld GPS devices, yielding a total of 46 data points. To minimize clustering effects that might skew the MaxEnt model’s results, the ENMtools package in R was employed for data screening. As a result, 18 final distribution points were retained for *D. flexicaule* (**Figure 2**).

#### 2.2.2 Data source processing

This research incorporated 36 environmental parameters, leveraging climatic datasets from the WorldClim platform (https://worldclim.org). The analysis encompassed bioclimatic indicators for the historical period (1970–2000) and projected future periods (2041–2060, 2061–2080) under multiple scenarios at a 30-second (~1 km) resolution. The 19 standard bioclimatic variables are labeled bio1–bio19. The climate projections used in this study were obtained from the Beijing Climate Center’s medium-resolution Climate System Model (BCC-CSM2-MR) (Tan et al., 2022), which is part of CMIP6. This model, recommended for use in China, encompasses three Shared Socioeconomic Pathways (SSPs)—SSP126, SSP245, and SSP585 (Zhou et al., 2019). Three topographic variables—elevation, slope, and aspect—were obtained from the Geospatial Data Cloud at a 90 m resolution, while 14 soil factors originated from the Harmonized World Soil Database (HWSD) at approximately 1 km resolution (https://www.fao.org/soils-portal/en/). The vector boundaries of Chinese administrative divisions (2020s) and 30-meter resolution land use classification data (2021s) were acquired from the Resource and Environmental Science and Data Center (RESDC), a leading repository for geospatial datasets in China. All datasets were reprojected to the WGS84 coordinate system and resampled to a uniform 1-km resolution to ensure consistency with our regional analytical framework. The administrative division data were sourced from the China Administrative Map database hosted by the Resource and Environment Science and Data Center (https://www.resdc.cn/). The copyright belongs to the platform and is provided under the license number GS (2024) 0650. All cartographic outputs were generated using Esri’s ArcMap platform (version 10.8.0).

**Figure 3:**
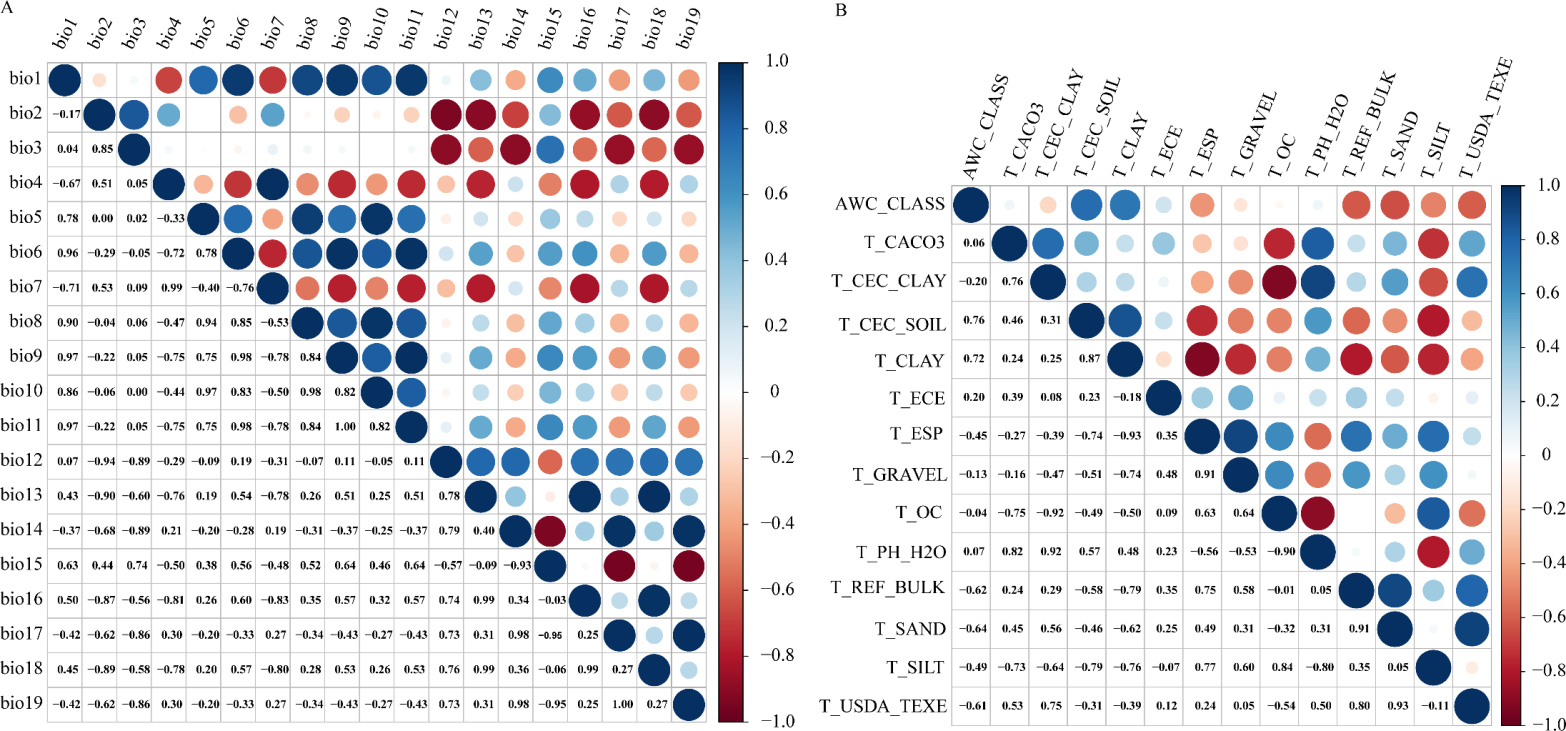
Spearman correlation analysis, Correlation analysis of Bioclimatic factors **(A)**, Correlation analysis of Soil factors **(B)**.

Because spatial autocorrelation among the 36 environmental factors (especially the 19 bioclimatic and 14 soil variables) could lead to overfitting (Sillero, 2011), a screening process was implemented to reduce multicollinearity. This involved a jackknife test and Spearman correlation analysis (**Figure 3**). Variables demonstrating inter-correlation values surpassing 0.8 were classified as exhibiting significant multicollinearity based on variance inflation criteria (Chhogyel et al., 2020). Through manual evaluation, factors with a 0% contribution in the jackknife test were eliminated, and where two variables correlated above 0.8, choose to remove variables with lower contribution rates. The final result is a set of four bioclimatic factors, six soil factors, and three terrain factors, with a total of 13 selected environmental variables (**Table 1**).

**Table 1:** Selected environmental factors.

Type	Abbreviation	Describe
Bioclimatic	Bio4	Temperature Seasonality
	Bio9	Mean Temperature of Driest Quarter
	Bio12	Annual Precipitation
	Bio14	Precipitation of Driest Month
Soil	AWC_CLASS	Available water capacity class
	T_REF_BULK	Topsoil Reference Bulk Density
	T_OC	Topsoil Organic Carbon
	T_CACO3	Topsoil Calcium Carbonate
	T_ESP	Topsoil Exchangeable sodium percentage
Topographic	Elevation	Elevation
	SLOPE	SLOPE
	Aspect	Slope orientation

### 2.3 Establishment and optimization of MaxEnt model

The MaxEnt model is a species distribution prediction method based on the principle of maximum entropy. It utilizes known species occurrence records and environmental variables as input data to estimate the probability of species occurrence under varying environmental conditions by maximizing entropy (Merow et al., 2013). MaxEnt modeling analysis was performed by importing the filtered set of 18 distribution points and 13 environmental factors in ACS format into the model. To characterize the available environmental space, we randomly generated 10,000 background points within the defined study area. The model was then run with 10 replicates, setting the maximum number of iterations to 10,000 to ensure full algorithm convergence (Chen et al., 2024). For each iteration of the experiment, a random subset comprising three-quarters of the data samples was allocated to model training, with the remainder designated as the evaluation set (Yuan et al., 2021). Model efficacy was assessed through analysis of receiver operating characteristic curves, where the area under the curve (AUC) served as the primary metric for quantifying predictive performance (Jiménez-Valverde, 2012). An AUC value of 1 signifies a perfect predictive capacity, whereas 0.5 implies a random prediction (Swets, 1988). For optimal performance, an AUC value above 0.9 is recommended (Sun et al., 2020). In addition, the Kappa coefficient (K) and True Skill Statistics (TSS) were employed to evaluate the predictive performance of the Maxent model (Cohen, 1960). A TSS value below 0.4 implies prediction failure. When the TSS value ranges from 0.4 to 0.55, it indicates fair predictive ability; values from 0.55 to 0.7 suggest moderate performance; those between 0.7 and 0.85 are regarded as good; Exceeding 0.85 indicates excellent performance (Allouche et al., 2006; Roberts et al., 2017). Likewise, for Kappa values, a value below 0.2 represents prediction failure. When the Kappa value is between 0.2-0.4, it indicates good performance; 0.4-0.6 is moderate; 0.6-0.8 is considered good; Exceeding 0.8 indicates excellence (Allouche et al., 2006; Roberts et al., 2017).

To assess the contribution of each environmental factor, the jackknife test in MaxEnt was employed (Shcheglovitova and Anderson, 2013). In MaxEnt, feature classes (FC) and the regularization multiplier (RM) are pivotal parameters that strongly influence prediction accuracy. Appropriate parameter calibration can markedly enhance the model’s overall performance (Radosavljevic and Anderson, 2014). We employed the ENMeval package in R for model calibration (Phillips and Dudík, 2008). Here, the RM was initialized at 0.5 and incremented in steps of 0.5 up to 5.0, yielding 10 iterations. We also tested various feature class combinations (L, LQ, H, LQH, LQHP, LQHPT), generating 60 parameter configurations in total. The model was then optimized based on these trials. The Akaike Information Criterion corrected for small sample sizes (AICc) is employed to evaluate model complexity and predictive performance. The model with the minimum AICc value (DAICc = 0) is generally identified as the most appropriate (Wen et al., 2024).

### 2.4 Suitable distribution areas of *D.flexicaule* with curved stems

To visualize the MaxEnt model’s predictive outcomes within the ArcGIS 10.8.0 platform, we implemented a four-tier classification system for *D. flexicaule* habitat suitability analysis. Based on the species’ stringent ecological requirements, suitability indices were partitioned as follows: unsuitable area (< 0.3), low suitable area (0.3-0.6), middle suitable area (0.6-0.8), and high suitable area (> 0.8).

Habitat suitability changes under current and future climatic conditions were assessed through spatial analyses conducted using SDMToolbox v2.5 (integrated within ArcGIS). This included quantification of suitable habitat area dynamics and centroid position shifts. The Geosphere package in R was subsequently employed to calculate centroid displacement distances between scenarios (Wang et al., 2023; Zhang et al., 2024).

### 2.5 Multivariate environmental similarity surface and most dissimilar variables analysis

Using the current climate layers as a reference (with approximate suitable areas delineated in ArcGIS), we applied the multivariate environmental similarity surface (MESS) approach to evaluate how future climate scenarios might alter *D. flexicaule* habitat suitability. MESS quantifies the degree of similarity (S) between current and future climate scenarios across corresponding regions. We identified the most dissimilar variables (MOD) to clarify key drivers of any projected distributional shifts.

MESS values range from 0 to 100, where S = 100 indicates future climate scenarios equivalent to the reference (i.e., no climatic discrepancy). Values between 0 and 100 signify varying degrees of divergence, while S<0 implies at least one bioclimatic variable lies beyond the reference range, marking a climate anomaly. We processed the results using MaxEnt Density and Novel tools (Elith et al., 2010; Li et al., 2016).

### 2.6 Conservation and development area of *D.flexicaule* with curved stems

Habitat quality refers to an environment’s capacity to support viable populations and stable communities over time. Fluctuations in habitat quality stem from factors such as location, topography, climate, and human activities (Mohan and Kandya, 2015). Evaluating habitat quality can thus indicate local biodiversity (Hillard et al., 2017) as well as gauge overall environmental integrity or degradation. The InVEST model, created through collaborative efforts between Stanford University, The Nature Conservancy (TNC), and the World Wildlife Fund (WWF), has become a widely utilized framework for evaluating ecological habitat quality in diverse land management scenarios. It accounts for vulnerability to threats, intensity of external pressures, and proximity-based spatial effects (Xu et al., 2023b).


$$Q_{xj}=H_{j}[1-(\frac{D_{xj}^{z}}{D_{xj}^{z}+k^{2}})]$$

The $Q_{xj}$ variable represents the quality of the habitat in grid z within habitat type j, with a value range of 0 to 1. A value close to 1 indicates a high-quality habitat with low maintenance costs and high biodiversity. The $H_{j}$ variable represents habitat suitability, while z represents the normalisation constant. The K variable represents the semi-saturation parameter, and the $D_{xj}$ variable represents the degree of habitat degradation.

Based on the InVEST user manual and related research (Bao et al., 2015; Hu et al., 2022; Xu et al., 2021). Threat factors quantify the disturbance intensity of land-use types on surrounding habitats; their weight values (range: [0,1]) reflect the destructive potential of the land-use type on habitats, with higher values indicating greater disruption. Sensitivity values (range: [0,1]) characterize the response level of land-use types to ecological threats, where values approaching 1 denote heightened sensitivity (Wu et al., 2020). We selected paddy fields, drylands, and urban land, among others, as threat factors, and we identified the most common threats to rice production, the weights of threat factors, the maximum influence distance and the attenuation type are assigned (**Tables 2**, **3**).

**Table 2:** Threat factor weight of study area.

Threat factor	Maximum impact distance(km)	Weight	Decay type
Paddyfield	4	0.7	linear
Dryland	3	0.5	linear
Urban	8	1	exponential
Village	5	0.6	exponential
other construction land	8	0.4	exponential
Unusedland	6	0.5	linear

**Table 3:** Sensitivity of land scape types to threat factors.

Land type	Habitat	Paddyfield	Dryland	Urban	Village	Other	Unusedland
Paddyfield	0.3	0	0.3	0.6	0.5	0.4	0.4
Dryland	0.3	1	0	0.6	0.5	0.5	0.4
Woodland	0.9	0.6	0.5	0.7	0.6	0.7	0.2
Shrub	1	0.6	0.6	0.8	0.7	0.7	0.2
Sparsewood	0.85	0.9	0.7	0.9	0.8	0.8	0.2
Other forest land	0.9	0.7	0.7	1	0.9	0.8	0.2
High coverage grassland	0.85	0.8	0.7	0.6	0.55	0.6	0.6
Medium coverage grassland	0.75	0.7	0.7	0.7	0.6	0.7	0.7
Low coverage grassland	0.7	0.6	0.7	0.8	0.7	0.8	0.8
Graff	1	0.8	0.65	0.85	0.7	0.5	0.3
Lake	0.9	0.3	0.3	0.75	0.6	0.6	0.3
Reservoir	0.7	0.7	0.7	0.85	0.7	0.5	0.3
Shoal	0.8	0.5	0.7	0.7	0.2	0.5	0.3
Urban	0	0	0.1	0	0	0	0
Village	0	0	0.1	0	0.5	0	0.1
Other	0	0	0	0.2	0.1	0	0
Swamp land	0.5	0.5	0.5	0.6	0.3	0.3	0.2
Unusedland	0	0	0	0	0	0	0

Finally, we integrated the InVEST model with the MaxEnt-derived suitability analysis, focusing on the most extensive and concentrated high-suitability regions of *D. flexicaule*. For better polygonal integrity and feasible subsequent analyses, we also included sections of moderate- and low-suitability areas. Such an approach effectively pinpoints priority conservation zones for *D. flexicaule* using limited resources while simultaneously designating practical development areas for industries tied to this medicinal species.

## 3 Result

### 3.1 MaxEnt model optimization results

**Figure 4:**
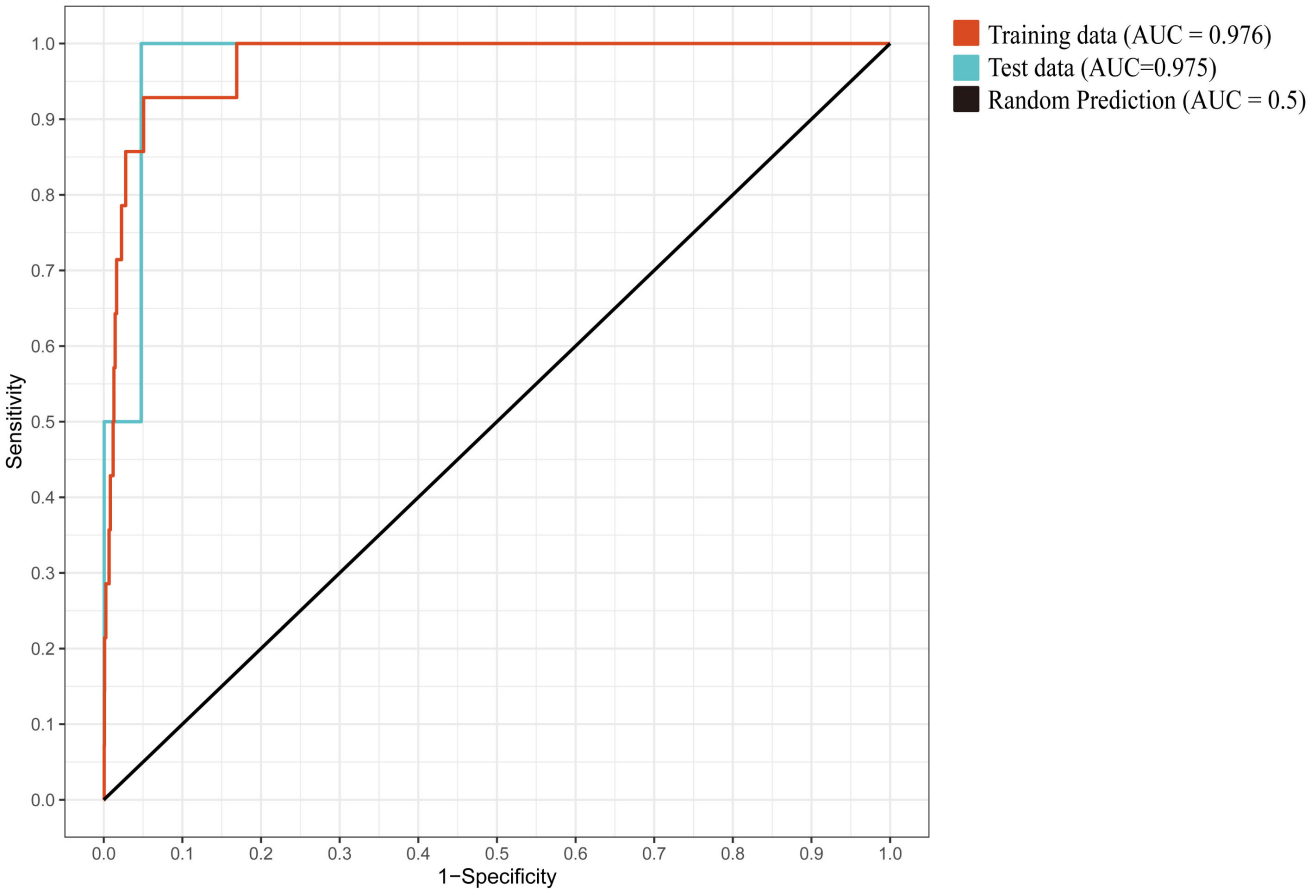
The mean ROC curve from 10 replicate training runs.

Under the default parameters, 10 replicate training runs yielded a mean AUC of 0.964. Based on the ENMeval package results (Chen et al., 2024), the optimal model was obtained when the feature class (FC) was set to LQ and the regularization multiplier (RM) was 1.5 (DAICc = 0). Under these conditions, the mean AUC across 10 replicate training runs was 0.976 (**Figure 4**), the Kappa coefficient (K) was 0.4379, and the True Skill Statistic (TSS) was 0.8522, indicating excellent predictive accuracy.

### 3.2 Current climate suitable area for *D.flexicaule*

**Figure 5:**
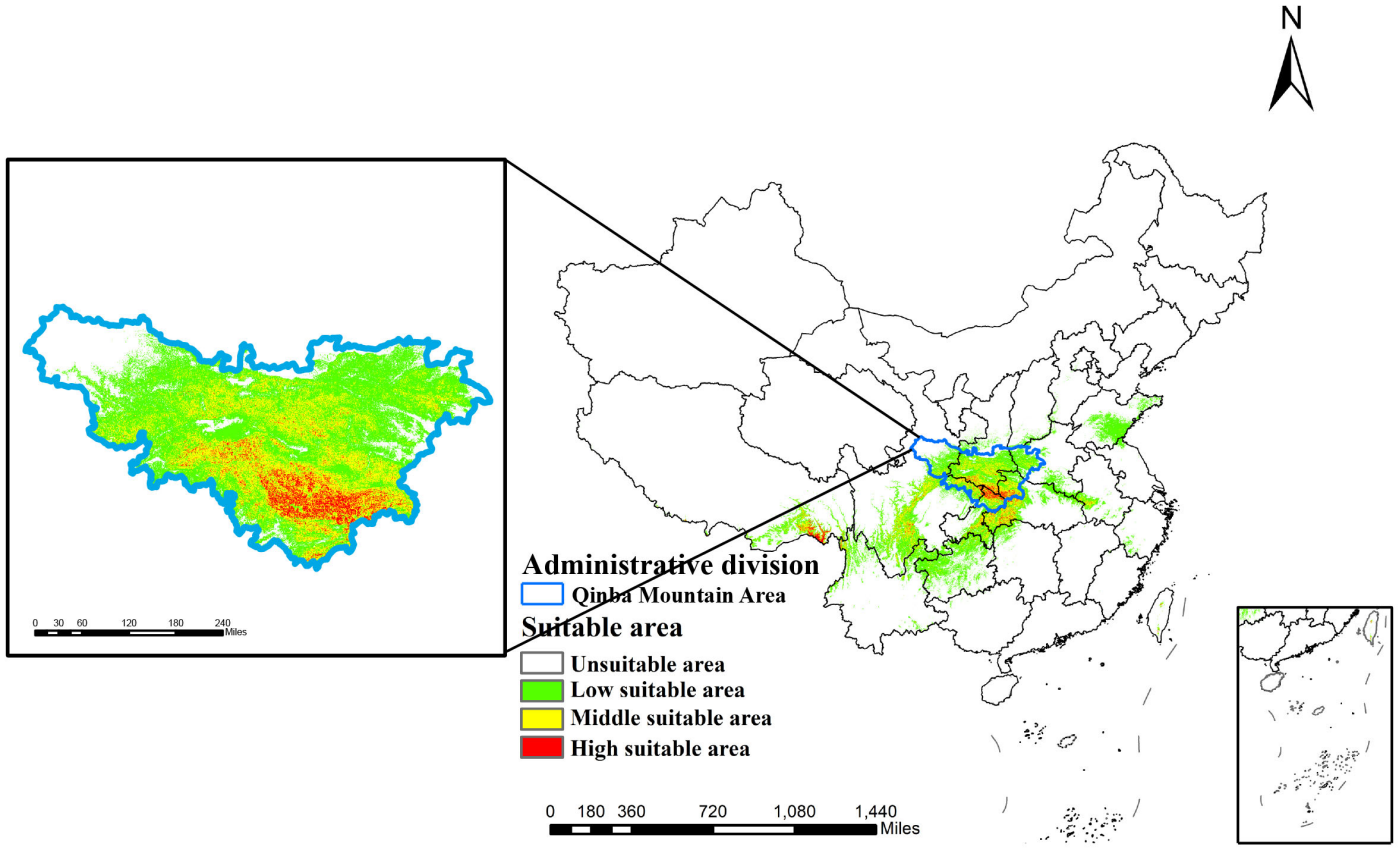
Current prediction of suitable areas for *D. flexicaule*. Map creation using ArcMap10.8.0 (URL: https://www.arcgis.com/index.html). China Administrative Map from Resource and Environmental Science Data Platform (https://www.resdc.cn/). GS (2024)0650.

Maxent’s predictions are visualized in ArcGIS to show the distribution of suitability for *D.flexicaule* current climate (**Figure 5**). Under present climate scenarios, *D. flexicaule* is mainly distributed in central China, including Sichuan, Chongqing, Henan and Hubei provinces. The total suitable area is around 64.3 × 10^4 km², which constitutes approximately 6.68% of China’s land area. Among these, the high-suitability zone measures about 2.4 × 10^4 km² (0.26% of China’s total land area), concentrated largely at the intersection of Sichuan, Shaanxi, and Hubei provinces, as well as in southeastern Tibet. In general, suitable distribution areas are mainly concentrated in the mountains around Sichuan Basin. MaxEnt predictions align well with the known geographic range of *D. flexicaule*, suggesting a reliable model.

### 3.3 Future climate suitable areas for *D.flexicaule*

**Figure 6:**
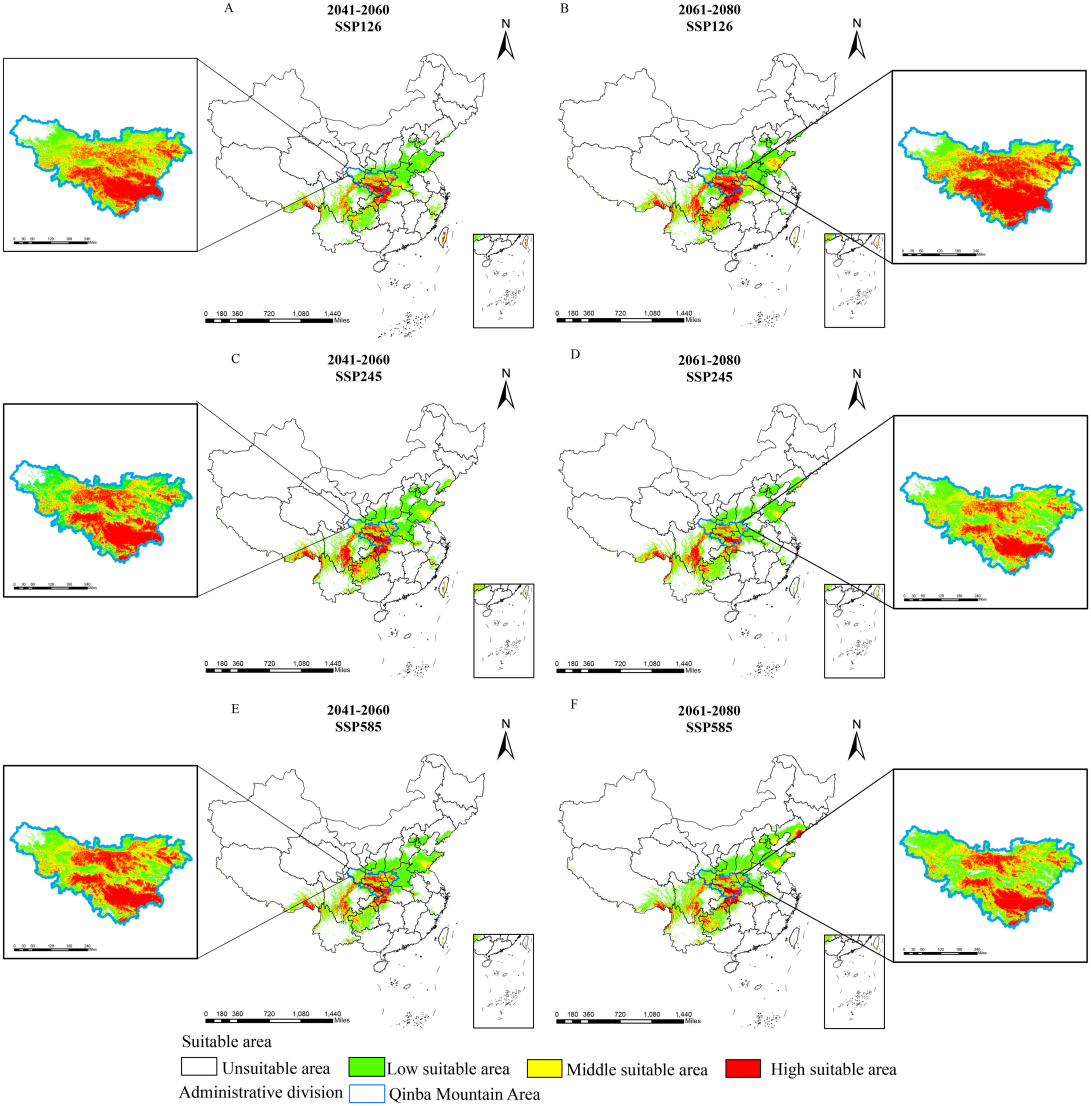
Prediction of suitable areas for *D. flexicaule* in the future [2041-2060: SSP126 **(A)**, 2041-2060: SSP245 **(C)**, 2041-2060: SSP585 **(E)**, 2061-2080: SSP126 **(B)**, 2061-2080: SSP245 **(D)** and 2061-2080: SSP585 **(F)**]. Map creation using ArcMap10.8.0 (URL: https://www.arcgis.com/index.html). China Administrative Map from Resource and Environmental Science Data Platform (https://www.resdc.cn/). GS (2024)0650.

We further generated future suitability maps for *D. flexicaule* in ArcGIS 10.2 (**Figure 6**). Future climate predictions indicate that the suitable areas for *D. flexicaule* will significantly expand (**Table 4**). The most pronounced expansion occurs under SSP585 in 2061–2080, where suitability grows from 64.3 × 10^4 km² to 159.04 × 10^4 km², an increase of 94.74 × 10^4 km² (147.34%). The smallest expansion appears under SSP1–2.6 in 2041–2060, yielding a total of 128.11 × 10^4 km², which is 63.81 × 10^4 km² (99.24%) larger than under current conditions.

**Table 4:** Suitable area under different climatic scenarios.

Period	Climate scenario	Low (x $10^{4}km^{2}$)	Moderately (x $10^{4}km^{2}$)	Highly (x $10^{4}km^{2}$)	Total (x $10^{4}km^{2}$)	Change rate(%)
1970-2000	Current	47.65	14.19	2.46	64.3	
2041-2060	SSP126	79.88	31.69	16.54	128.11	99.24
	SSP245	93.03	32.66	25.79	157.38	144.76
	SSP585	85.11	32.25	17.74	135.5	110.73
2061-2080	SSP126	75.78	40.24	30.04	146.06	127.15
	SSP245	83.65	31.92	18.23	133.8	108.09
	SSP585	98.36	40.29	20.39	159.04	147.34

Under all scenarios, the total extent of low-, moderate-, and high-suitability zones rises. Under SSP585 in 2061–2080, low- and moderate-suitability areas exhibit the most robust growth, whereas high-suitability zones expand most under SSP126 in 2061–2080. These findings imply that *D. flexicaule* habitat is highly responsive to climatic shifts, regardless of the scenario.

### 3.4 Dynamic changes of suitable areas for *D. flexicaule* in different periods

**Figure 7:**
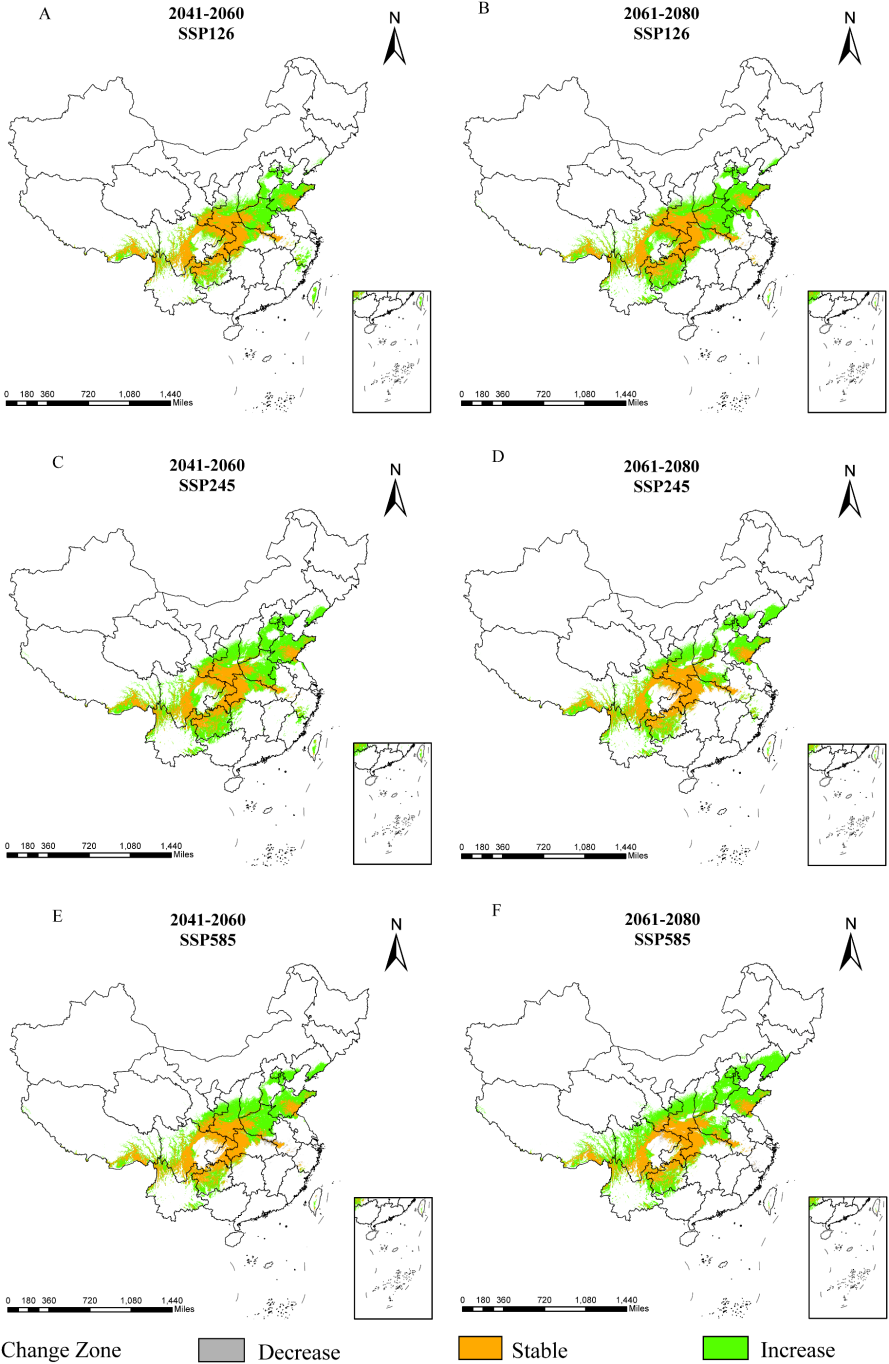
Dynamic changes of *D*. *flexicaule* under future climate conditions [2041-2060: SSP126 **(A)**, 2041-2060: SSP245 **(C)**, 2041-2060: SSP585 **(E)**, 2061-2080: SSP126 **(B)**, 2061-2080: SSP245 **(D)** and 2061-2080: SSP585 **(F)**]. Map creation using ArcMap10.8.0 (URL: https://www.arcgis.com/index.html). China Administrative Map from Resource and Environmental Science Data Platform (https://www.resdc.cn/). GS (2024)0650.

Compared with current conditions, *D. flexicaule’s* suitable distribution areas vary across climate scenarios and time periods (**Table 5**; **Figure 7**). Under SSP245 for 2041–2060, the smallest decline in suitable area is observed: a reduction of 0.17 × 10^4 km² (0.26%). The largest decrease occurs under SSP585 for 2041–2060, amounting to 1.72 × 10^4 km² (2.67%). By contrast, the most pronounced increase occurs under SSP585 for 2061–2080, with a 96.24 × 10^4 km² (149.67%) expansion, while the smallest rise takes place under SSP126 for 2041–2060, totaling 64.73 × 10^4 km² (100.67%).

**Table 5:** Current and future changes in suitable areas for *D. flexicaule*.

Period	Climate scenario	Habitat area (x $10^{4}km^{2}$)	Decrease (x $10^{4}km^{2}$)	Stable(x $10^{4}km^{2}$)	Increase (x $10^{4}km^{2}$)	Parentage low(%)	Parentage gain(%)
Current		64.3					
2041-2060	SSP126	128.11	0.92	63.38	64.73	1.43	100.67
	SSP245	157.38	0.17	64.13	93.25	0.26	145.02
	SSP585	135.5	1.72	62.58	72.92	2.67	113.41
2061-2080	SSP126	146.06	0.5	63.8	82.26	0.78	127.93
	SSP245	133.8	0.59	63.71	70.09	0.92	109.00
	SSP585	159.04	1.5	62.8	96.24	2.33	149.67

As shown in **Figure 7**, unchanged areas are predominantly located in Sichuan, Chongqing, Shaanxi, Hubei, and Shandong, whereas regions exhibiting decreases are concentrated in Anhui and Hubei. Areas displaying increases are primarily found in Shandong, Shanxi, Taiwan, Hebei, Yunnan, and Tibet. Overall, *D. flexicaule*’s habitat suitability responds markedly to climate change, with broadly consistent patterns across similar climate scenarios in different periods. Although all future scenarios point to an overall expansion of suitable habitat, the magnitude of change varies among them.

**Figure 8:**
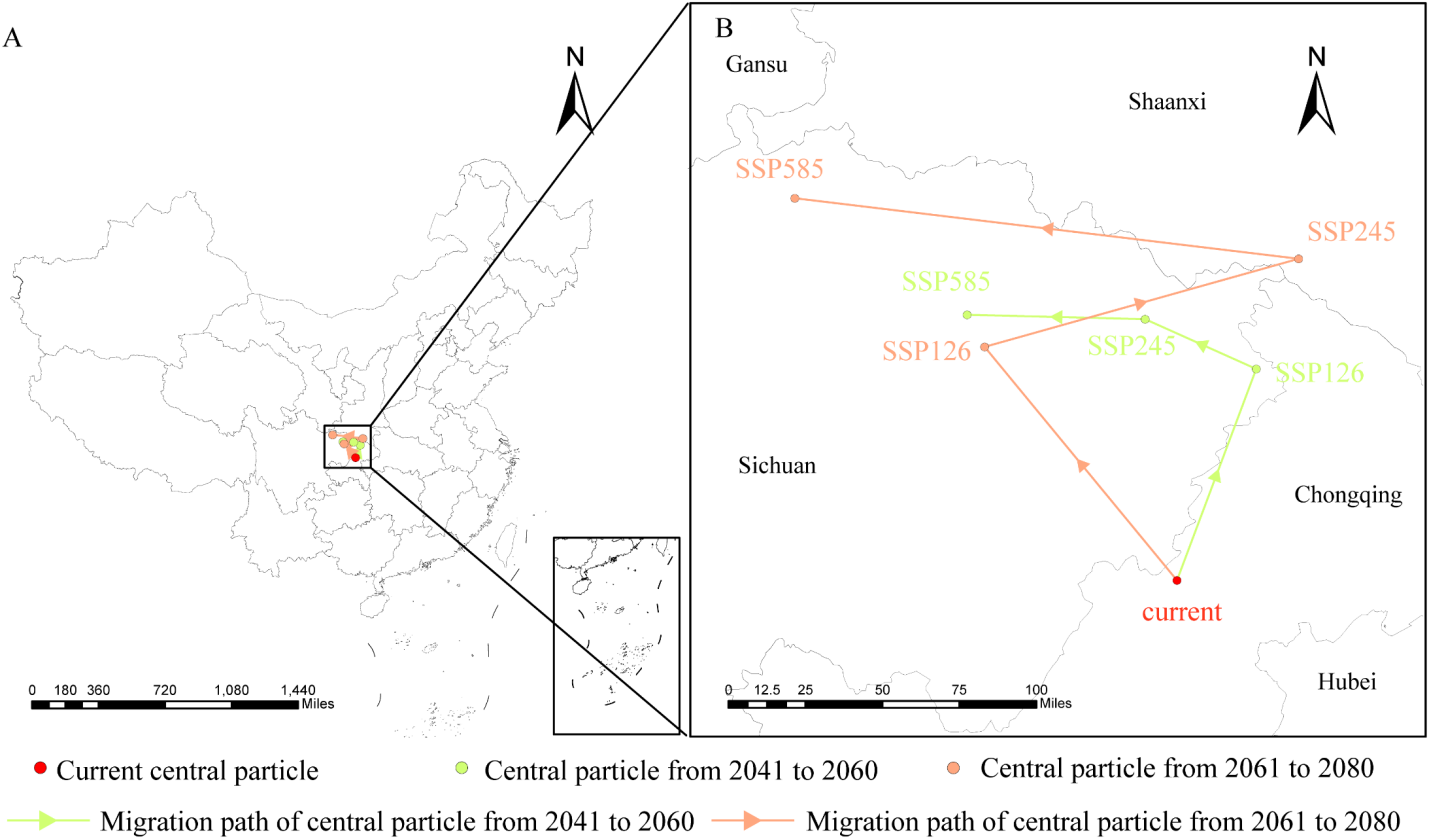
Geographical changes of the central particle in *D. flexicaule* under different climatic scenarios and periods [**(B)** is an enlargement of the part of **(A)**].

From a spatial perspective (**Figure 8**), the centroid of *D. flexicaule*’s suitable habitat shifts in multiple directions but trends northwestward overall. Under current conditions, the centroid is located near Yujia Town in Wanzhou District, Chongqing (E 107°57′38.82″, N 30°46′28.18″). Under SSP245 for 2041–2060, it moves 137.83 km to the southwest, landing in Wanyuan City, Dazhou, Sichuan (E 107°44′41.68″, N 31°59′55.84″). Under SSP585 for 2061–2080, it shifts 282.29 km to the northwest, reaching Chaotian District, Guangyuan, Sichuan (E 105°46′46.80″, N 32°30′5.69″). The results indicate that future changes in precipitation and temperature will push the suitable habitat center of *D. flexicaule* towards the northwest to adapt to the impacts of future climate.

### 3.5 Multivariate environmental similarity surface and most dissimilar variable analysis

**Figure 9:**
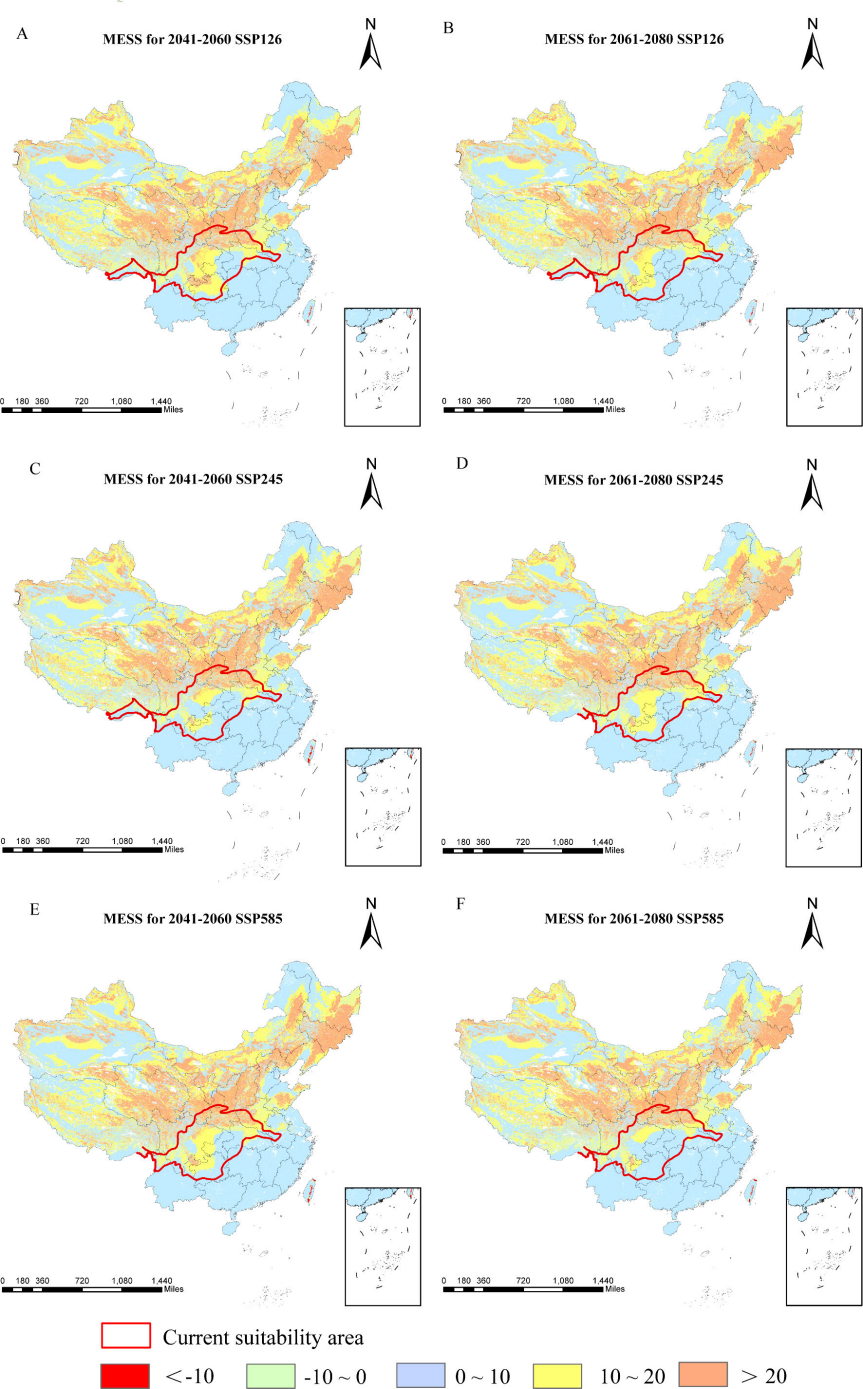
Multivariate environmental similarities of *D*. *flexicaule* [2041-2060: SSP126 **(A)**, 2041-2060: SSP245 **(C)**, 2041-2060: SSP585 **(E)**, 2061-2080: SSP126**(B)**, 2061-2080: SSP245 **(D)** and 2061-2080: SSP585 **(F)**]. Map creation using ArcMap 10.8.0 (URL: https://www.arcgis.com/index.html). China Administrative Map from Resource and Environmental Science Data Platform (https://www.resdc.cn/). GS (2024)0650.

**Figure 10:**
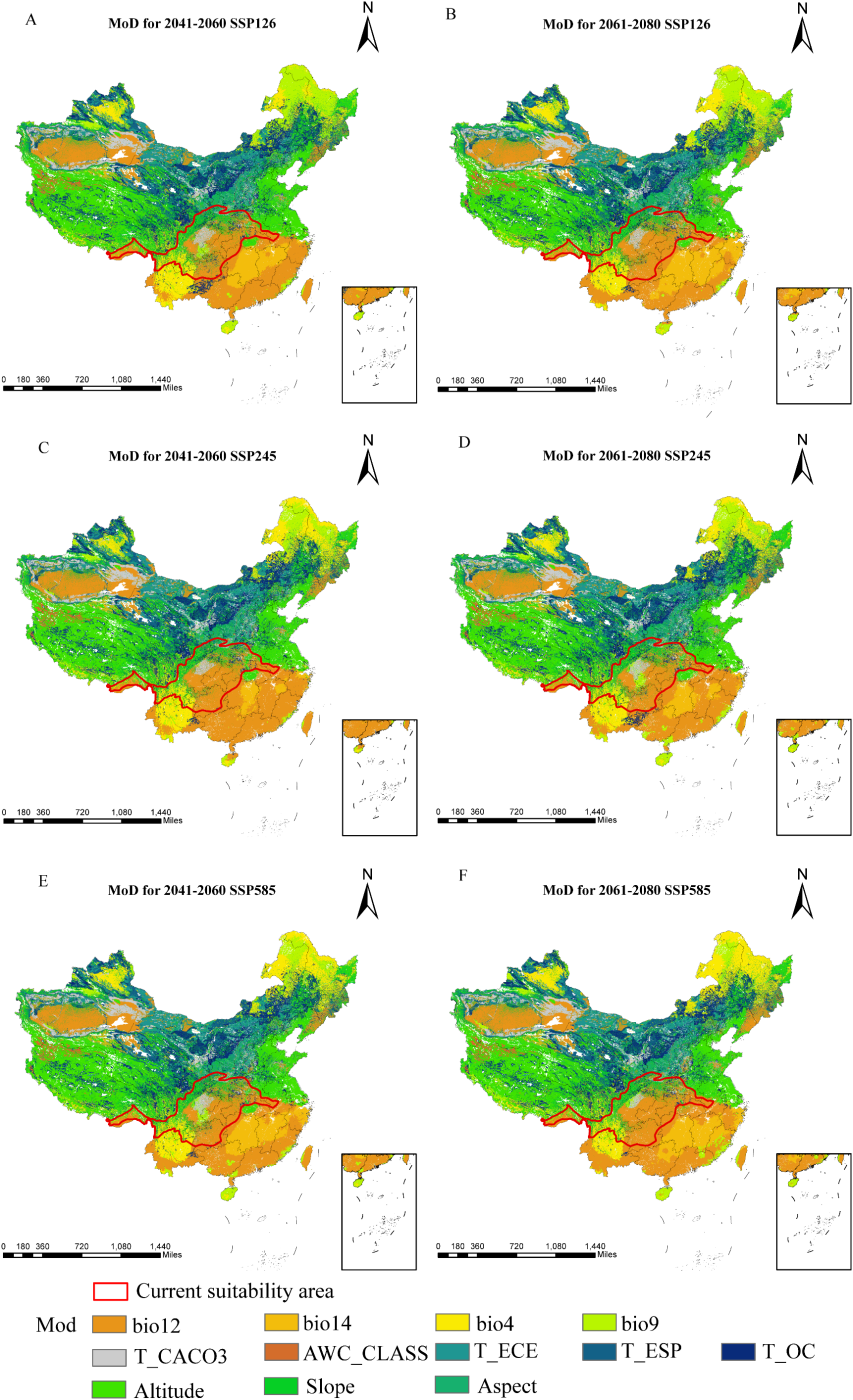
Most Dissimilar Variables of *D*. *flexicaule* [2041-2060: SSP126 **(A)**, 2041-2060: SSP245 **(C)**, 2041-2060: SSP585 **(E)**, 2061-2080: SSP126 **(B)**, 2061-2080: SSP245 **(D)** and 2061-2080: SSP585 **(F)**]. Map creation using ArcMap10.8.0 (URL: https://www.arcgis.com/index.html). China Administrative Map from Resource and Environmental Science Data Platform (https://www.resdc.cn/). GS (2024)0650.

Relative to current conditions, the mean multivariate similarity across future climate scenarios ranges from 11.53 to 12.29, indicating an overall positive environmental similarity. During 2061–2080, SSP245 displays the highest multivariate similarity and the lowest degree of climatic anomaly, whereas SSP585 shows the lowest similarity and thus the greatest climate deviation (**Figure 9**). The variables most responsible for changes in suitable regions are annual precipitation, elevation, and precipitation in the driest month (**Figure 10**). Substantial changes in the mean temperature of the driest quarter appear mainly under SSP126 and SSP585 (2041–2060) and SSP245 (2061–2080). Overall, precipitation factors exert the strongest influence, followed by topography and temperature, while soil variables are sparse and contribute the least.

### 3.6 The main environmental factors affecting the distribution of *D.flexicaule*

**Figure 11:**
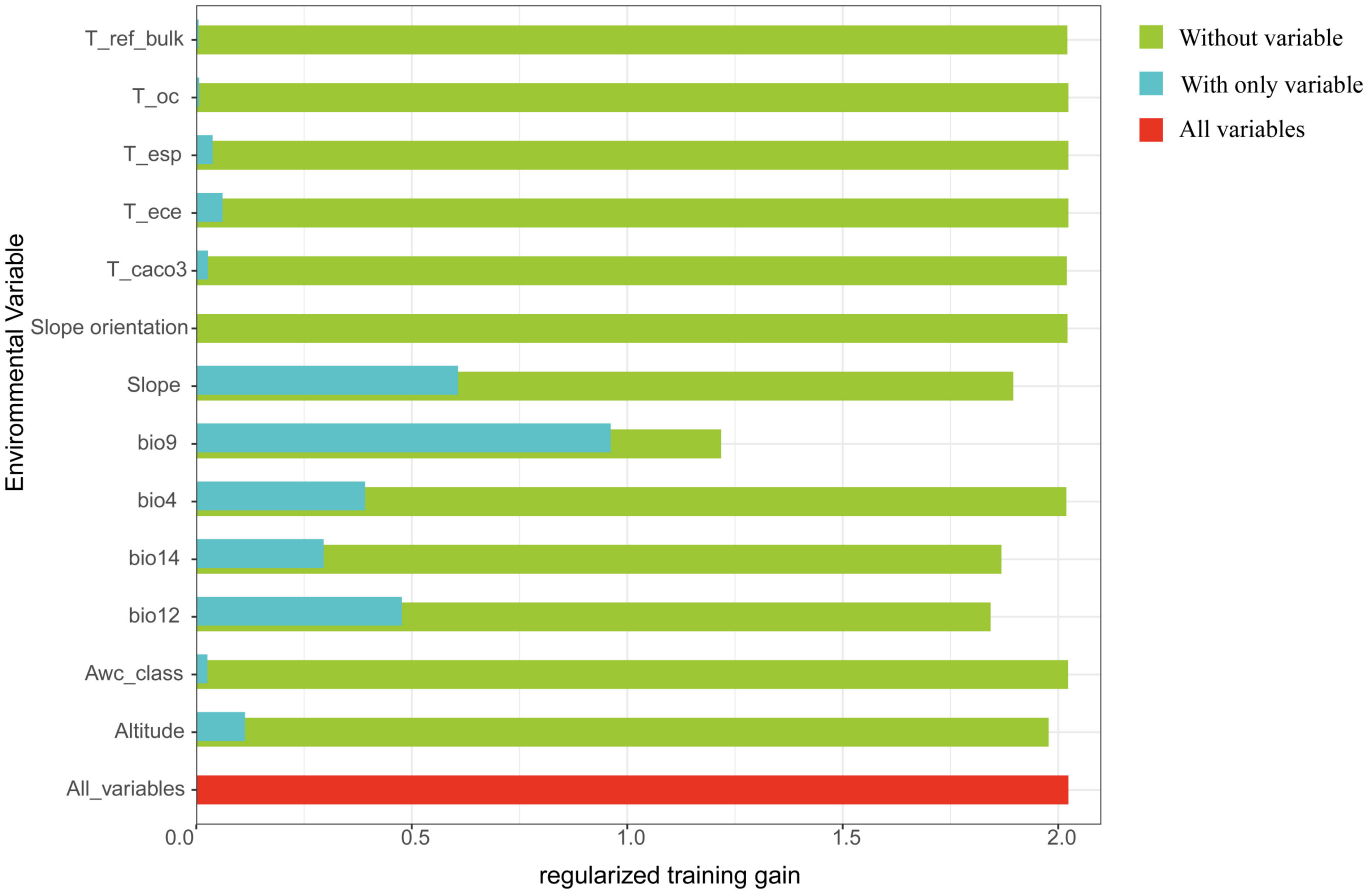
Regularized training gain (Green represents without variable, blue represents without only variable, and red represents all variables).

According to MaxEnt projections, the five leading determinants of *D. flexicaule* distribution are the mean temperature of the driest quarter (43.7%), slope (26.4%), annual precipitation (11.5%), elevation (7.4%), and precipitation in the driest month (7.0%). In the jackknife test (**Figure 11**), the mean temperature of the driest quarter exhibits the highest normalized training gain when used alone, implying it holds the most predictive information, followed by slope and annual precipitation. Notably, excluding the driest-quarter temperature from the model yields the lowest training gain, underscoring its unique importance. Hence, the driest-quarter temperature emerges as the critical driver behind the distribution of *D. flexicaule*.

**Figure 12:**
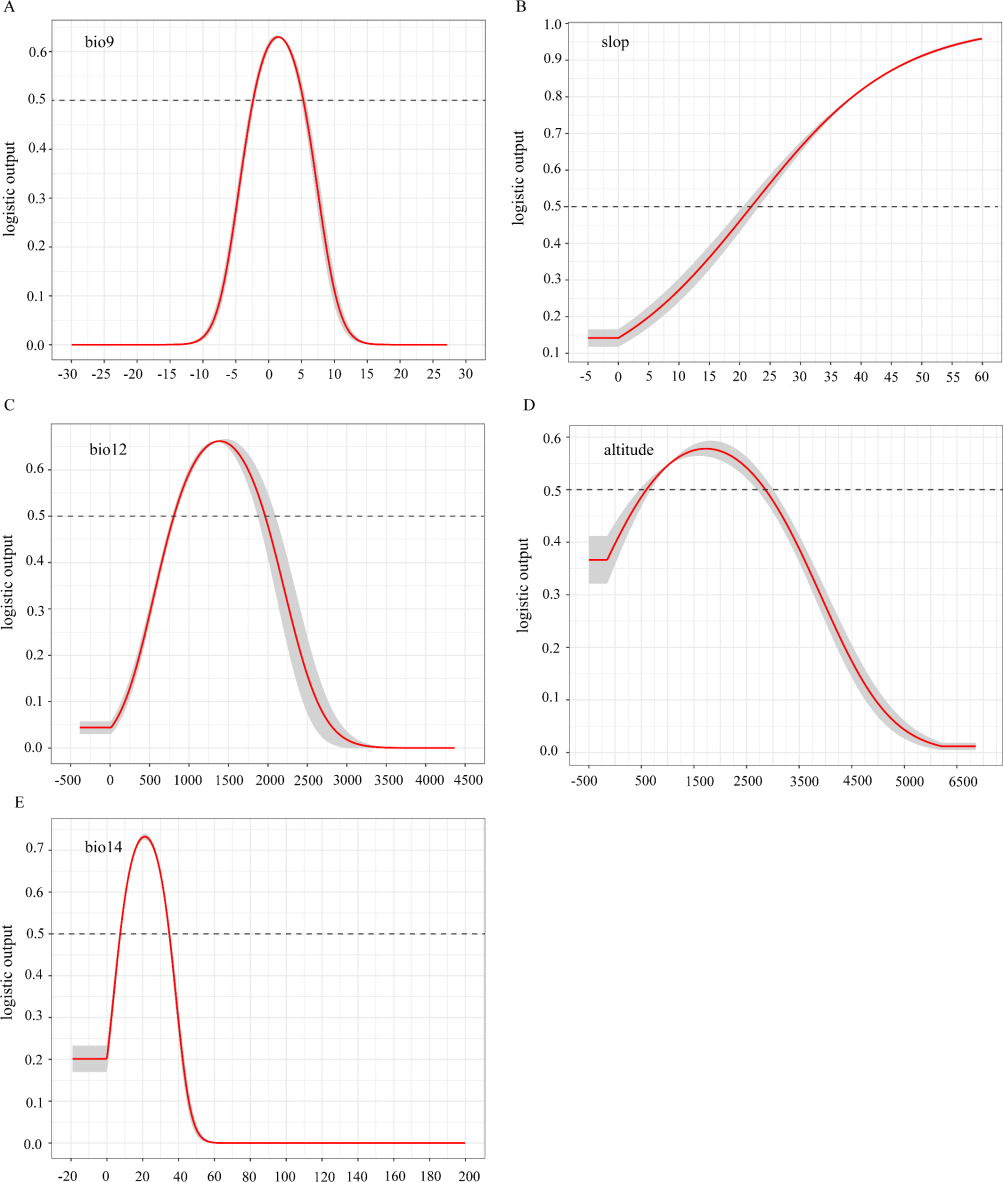
Single factor response curve of the current climate [**(A)** bio 9, **(B)** slop, **(C)** bio 12, **(D)** altitude, **(E)** bio 14].

Response curves for these five key variables (**Figure 12**) show that when each factor’s survival probability surpasses 0.5, the corresponding range is suitable for growth. Specifically, *D. flexicaule* has a survival probability under 0.1 at driest-quarter temperatures below -7.5°C, rising steadily to its peak near 1.5°C, then declining again as temperatures exceed that point. A probability above 0.5 persists between roughly -2.5°C and 5.0°C. Under current conditions, the model deems the following ranges suitable: a driest-quarter mean temperature of -2.5°C to 5.0°C, slopes steeper than 22°, annual precipitation between 780 mm and 1,900 mm, elevations of 600–2,800 m, and driest-month precipitation of 8–35 mm.

### 3.7 Priority protection and development areas for *D.flexicaule* with curved stems

**Figure 13:**
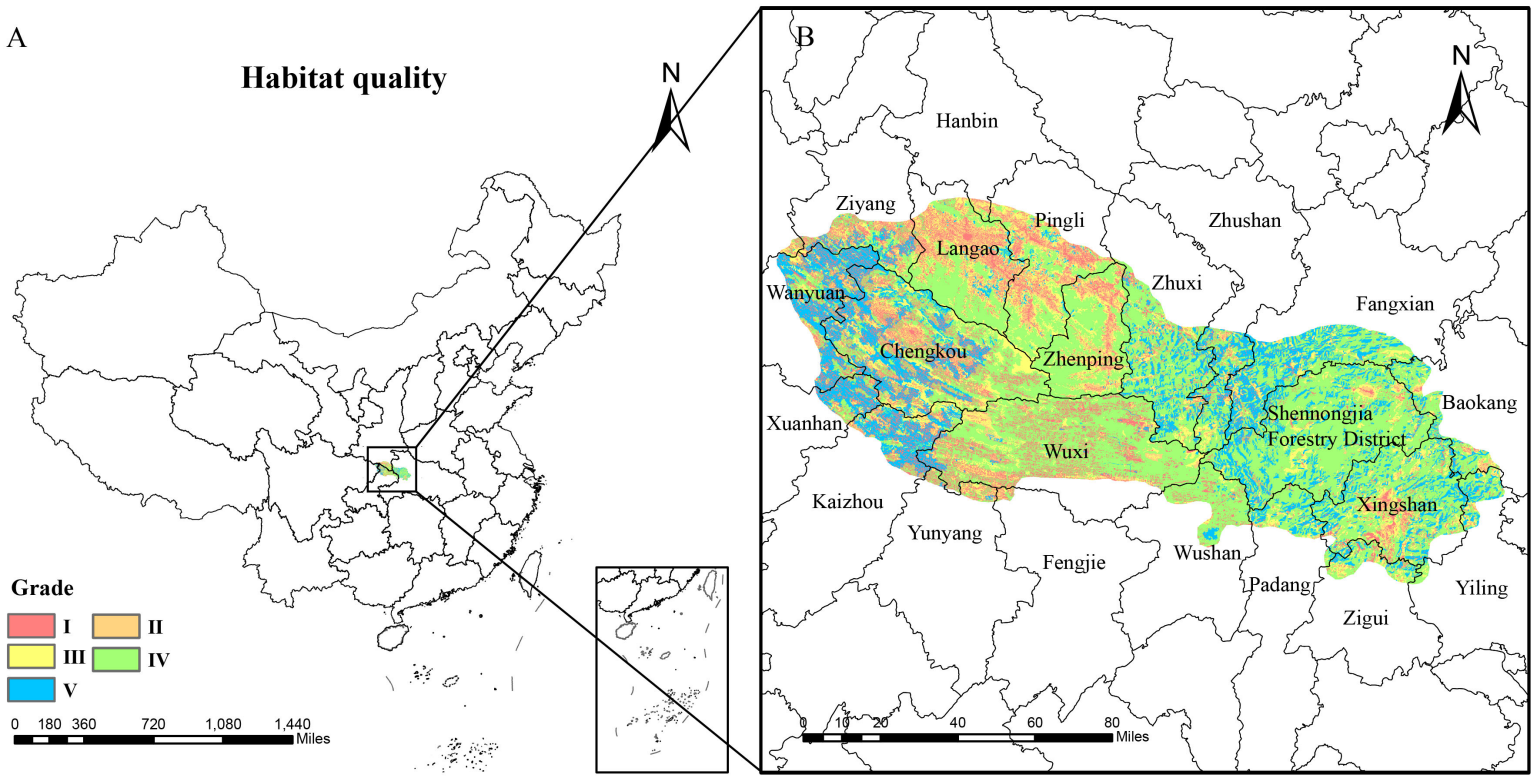
Habitat quality in some highly suitable areas [**(B)** is an enlargement of the part of **(A)**]. Map creation using ArcMap10.8.0 (URL: https://www.arcgis.com/index.html). China Administrative Map from Resource and Environmental Science Data Platform (https://www.resdc.cn/). GS (2024)0650.

Using the InVEST model to calculate habitat quality in some suitable areas for *D.flexicaule*, the results were imported into ArcGIS, and the habitat quality was classified into five grades using the natural breaks classification method (**Figure 13**): poor (P < 0.475), suboptimal (0.475 ≤ P < 0.702), moderate (0.702 ≤ P < 0.848), good (0.848 ≤ P < 0.922), and excellent (P ≥ 0.922). In general, the better the habitat quality and the greater the biodiversity, the better the ecological suitability of the area. Therefore, applying limited conservation resources to the most valuable areas can achieve optimal conservation outcomes. This study designates areas with excellent habitat quality as priority conservation areas. Considering the need for industrial development, especially in recent years with increasing demand for product quality, authenticity, and rural revitalization, areas with moderate and good habitat quality are designated as priority development areas. On the one hand, high habitat quality can ensure product quality. On the other hand, the construction of ecological industrial bases, combined with the ornamental value of *D.flexicaule*, can promote the development of ecological tourism in these areas. As shown in the **Figure 13**, the priority conservation areas for *D.flexicaule* are primarily concentrated in the southern foothills and the eastern end of the Qinba Mountains, including locations such as Wanyuan City and Xuanhan County in Sichuan Province, Chengkou County in Chongqing, and Fangxian County. The priority development areas for *D.flexicaule* are mainly located in the northern foothills of the Qinba Mountains in Chengkou County, Chongqing, as well as in Xingshan County, Hubei Province, and Zhenping County, Shaanxi Province. The results of this study align with the conservation and development areas for *D.flexicaule* in the Qinba Mountain region. Moreover, both the priority conservation and development areas are relatively concentrated, which is beneficial for both species conservation and the development of industries related to the species.

## 4 Discussion

### 4.1 MaxEnt model evaluation

This study used the Maxent model for modeling, which predicted species distribution based on species formation records and environmental factors. While most researchers use the default parameters for modeling with MaxEnt, some have noted that the default parameters may lead to overly complex models, making the results difficult to interpret and not always suitable for species distribution modeling (Merow et al., 2013; Radosavljevic and Anderson, 2014). Aiming at the overfitting problem of the model, this study uses the “ENMeval” package in R to optimize the default parameters of the model. The “kuenm” package in R (http://github.com/marlonebos/kuenm) offers similar functionality by integrating with the MaxEnt model to automate the modeling workflow. It systematically simulates various combinations of feature classes (FC) and regularization multipliers (RM), generating and evaluating multiple candidate models to identify the optimal parameter settings (Cobos et al., 2019; Yao et al., 2024). Considering the potential spatial autocorrelation among distribution points, this study utilized the ENM Tools package in R to filter the points. The software package stochastically removes duplicate points within identical grid cells based on the spatial resolution of climatic factors, ensuring that each grid cell retains a singular representative data point. Other researchers (Liu et al., 2021; Yang et al., 2022) also utilized proximity analysis within GIS geospatial processing tools to retain distribution points closest to each grid centroid, achieving favorable outcomes.

Contemporary ecological modeling increasingly incorporates multidimensional environmental variables such as bioclimatic, topographic, and soil factors, which have demonstrated enhanced predictive performance in species distribution analyses (Jing et al., 2020; Wei et al., 2020). However, strong correlations among these variables, particularly in biological and soil datasets, can affect modeling results. Accordingly, we employed a jackknife test and Spearman correlation analysis to identify the most relevant variables, thereby enhancing the model’s accuracy. Though MaxEnt offers advantages over other species distribution models, it has limitations (Che, 2022). First, the model forecasts a species’ maximum potential distribution, which may not fully coincide with the species’ observed range (Ta et al., 2021). Second, the niche-based foundation of MaxEnt presupposes unlimited expansion under ideal conditions, whereas in reality, species distributions are constrained by various factors beyond the model’s scope.

### 4.2 Important variables of *D.flexicaule*

Understanding how a species’ geographic distribution correlates with environmental variables is a critical first step in conservation (Harapan et al., 2022). Climate change often affects the physiological and biochemical characteristics of plants in complex ways, often leading to changes in their suitable habitats (Cao et al., 2016; Li et al., 2020b). Under the current climate scenario, temperature, slope and precipitation are the main factors affecting *D. flexicaule* distribution was the main variable, while the influence of soil factors was relatively small. The temperature exerts a paramount regulatory influence on the growth, physiological development, and metabolic processes of *Dendrobium* species (Orchidaceae). (Taticharoen et al., 2023). Studies have indicated that the normal growth and fruiting of *D. flexicaule* require an average annual temperature ranging from 8 to 15°C, along with substantial water availability. In particular, the Funiu Mountain area receives approximately 900 mm of average annual rainfall (Zhang et al., 1999). According to the response curves of each factor predicted by the MaxEnt model, the optimal range for *D.flexicaule* is a mean temperature of 2°C during the driest quarter, a steep slope, and annual precipitation of 1350 mm. This aligns with the species’ biological preference for warm, steep, and humid regions (Zhang, 2016). The distribution models of orchids revealed that Bio17, Bio12, and Bio4 were the key climatic determinants. This indicates that these parameters significantly shape the spatial patterns of orchid habitats, with closely related species demonstrating analogous adaptation thresholds to bioclimatic constraints (Zheng et al., 2023).

### 4.3 Regional distribution of suitability of *D.flexicaule*

Species’ responses to climate change often hinge on the interplay between habitat suitability and geographic shifts (Davis, 2001). Many taxa are anticipated to move toward higher latitudes and elevations in response to warming, though the extent and direction of these relocations vary markedly across species (Chen et al., 2011). Studies aimed at pinpointing orchid-rich zones for conservation—such as southeastern Tibet, western Yunnan, and central Yunnan—demonstrate partial overlap with the distribution of *D. flexicaule* (Zhan et al., 2024), and modeling efforts similarly highlight that suitable areas for *D. flexicaule* cluster in Sichuan, Chongqing, Henan, Hubei, Tibet, and Yunnan (Sun et al., 2020). However, its high suitability area is extremely limited, covering only 2.46 × 10^4 km². *D. flexicaule* exhibits stringent microclimatic requirements: it is confined to near-vertical, moss-covered limestone cliffs or rock crevices that are simultaneously warm-cool, persistently moist yet free of standing water, irradiated by diffuse light but shielded from direct insolation, and subject to continuous gentle ventilation without mechanical wind stress; these microhabitats are typically characterised by the audible presence of running water while surface water remains absent (Zhang, 2016). Such stringent microclimatic requirements explain the extremely limited extent of climatically suitable habitat predicted by niche models.

In parallel, our projections for *D. flexicaule* reveal considerable habitat changes under future warming, with all levels of suitability expanding; high-suitability areas show the largest increase, while lower-suitability areas show the smallest. This pattern underscores *D. flexicaule’s* strong sensitivity to global temperature rises. Spatially, *D. flexicaule* habitat consistently broadens in response to warming. Mycorrhizal fungi play a crucial role in buffering the extinction risk of plant hosts. They can either promote or hinder the dispersal success of plant migration from harsh environments, and by buffering host plants, they help reduce extinction risk and facilitate adaptation to new climates (Bennett and Classen, 2020). Fernandez et al. (2023) found that warming and reduced rainfall significantly altered the community composition of ectomycorrhizal fungi (EMF), while Wu et al. (2023) reported that warming significantly enhanced the photosynthetic rate and growth of plants associated with endophytic mycorrhizal fungi. Therefore, we hypothesize that future temperature changes may influence mycorrhizal fungi, thereby affecting the germination rate of *D. flexicaule* (Royal Botanic Gardens, Kew, 2025, internal report). Multivariate environmental similarity analyses suggest that future climate anomalies shift to lower and higher latitudes, driven primarily by precipitation variables (e.g., total annual precipitation, precipitation in the driest month). Meanwhile, scenarios with the most pronounced expansions in suitable habitat also exhibit the largest changes in the mean temperature of the driest quarter, identified here as the most critical limiting factor. *D. flexicaule* thrives in warm, humid conditions; thus, rising temperatures make additional regions habitable. Overall, the marked increase in suitable habitat indicates that *D. flexicaule* could be cultivated in its current range without extensive damage from future climate change.

Nevertheless, China’s warming rate already exceeds the global average (Center), and escalating temperatures are projected to intensify the frequency of extreme weather events (Wang et al., 2017). Such events—including heatwaves, cold snaps, and erratic rainfall—have substantially affected natural ecosystems (Yu et al., 2023; Piao et al., 2019). As a core component of terrestrial ecosystems, vegetation is especially vulnerable to these phenomena (Liu et al., 2023). Plants typically occupy narrow climate envelopes conducive to their growth; when these thresholds are exceeded, growth rates diminish (Schlenker and Roberts, 2009; Hoffman et al., 2018) or, in severe cases, populations may perish (Luo, 2011). In the context of global change, epiphytic plants appear to be particularly sensitive and more vulnerable than other terrestrial plants (Gentry and Dodson, 1987; Kreft et al., 2004). While model results suggest *D. flexicaule* could benefit overall from a warmer climate, the surge in extreme weather—particularly harmful for this climate-sensitive and endangered species—cannot be ignored. Indeed, sudden cold snaps can severely damage *D. flexicaule*, and temperatures below -10°C for extended periods can cause varying degrees of frost injury (Zhang, 2016). Moreover, cultivated plants frequently exhibit lower stress tolerance, placing them at higher risk of large-scale mortality under extreme conditions.

A variety of approaches exist for classifying suitable areas, including manual classification (Yan et al., 2021), the IPCC’s probability thresholds (Manning, 2006), intervals derived from top 80% and bottom 20% values (Zhu et al., 2023), and the widely used natural breaks method in ArcGIS (Wang et al., 2023; Ho and Umitsu, 2011). However, we found the results from the natural breaks method differed substantially from observed realities. Consequently, manual classification was ultimately selected to delineate *D. flexicaule’s* suitable habitat in a manner better aligned with its actual distribution (Wang et al., 2023).

### 4.4 Priority protection and development areas for *D.flexicaule* with curved stems

Land-use changes substantially impact species distributions (Cardinale et al., 2012). Many studies relying on MaxEnt limit environmental factors to bioclimatic, soil, and topographic data, often overlooking land-use types. Yet habitat quality is especially critical for species like *D. flexicaule*, which have precise environmental needs. While some researchers (Chao et al., 2021) incorporate land-use factors, these variables are sometimes excluded or underrepresented during variable selection.

Conservation efforts aim to preserve biodiversity—particularly for endangered plants—and ensure its sustainable use (Adhikari et al., 2019). Habitat loss is a principal driver of biodiversity decline, making habitat protection vital (Françoso et al., 2015). By integrating the MaxEnt and InVEST models, our study identifies key areas for *D. flexicaule* conservation and development. Our findings reveal that priority conservation zones are concentrated in Wanyuan City and Xuanhan County (Sichuan), Chengkou County (Chongqing), and Fang County and the Shennongjia Forestry District (Hubei). Priority development areas lie mainly in Chengkou County (Chongqing), Xingshan County (Hubei), and Zhenping County (Shaanxi). These counties sit in the Qinba Mountains, a region with varied landforms and one of China’s major biodiversity hotspots (Qiao et al., 2016). The Qinba Mountains, in particular, feature mild climates, ample sunlight, abundant rainfall, and sufficient heat—conditions that favor cultivating medicinal plants (Zhang et al., 2008).

Based on the research findings, the following integrated recommendations are proposed to enhance practical application and policy relevance: In priority conservation areas characterized by excellent habitat quality (e.g., the southern foothills and eastern Qinba Mountains, including specific locations such as Wanyuan City, Chengkou County, and Fangxian County), these zones should be formally incorporated into ecological protection redlines, with strict restrictions on large-scale infrastructure and mining activities, supported by eco-compensation mechanisms to balance conservation objectives and local livelihoods. In priority development areas with moderate to good habitat quality (e.g., northern Chengkou County, Xingshan County, and Zhenping County), community-led initiatives such as sustainable cultivation of *D. flexicaule* and eco-certification programs should be promoted, capitalizing on the species’ ornamental value to develop ecological tourism and support rural revitalization. Furthermore, a dynamic monitoring system integrating GIS and remote sensing technologies is recommended to regularly track habitat changes in both types of zones, enabling adaptive management and coordinated conservation-development outcomes.

### 4.5 Limitation

Plants’ adaptation to the environment and their dispersal capabilities often determine their distribution patterns, while both biotic and abiotic factors can also influence these patterns. Although water and temperature conditions primarily define *D. flexicaule* habitat suitability, unaccounted-for soil and biotic interactions may also play roles. As an epiphytic plant, the growth of *D.flexicaule* is also influenced by the specific characteristics of its host epiphytes (Wang, 2024). The absence of future soil and topographic changes in our projections could therefore introduce some bias into the results.

Therefore, to formally incorporate an assessment of these potential biases into our discussion, we acknowledge key uncertainties stemming from our model’s static treatment of soil properties and future land-use patterns. Soil characteristics influence epiphyte microhabitats by affecting host tree bark chemistry (Tatsumi et al., 2023), and their constancy in projections may introduce inaccuracies. Similarly, by omitting dynamic land-use change (e.g., deforestation), our model fails to account for potential habitat fragmentation and loss that could directly impact the availability of host trees, irrespective of climatic suitability (Jantz et al., 2014).

To quantify these potential biases, we recommend future studies employ sensitivity analysis (e.g., Sobol’ indices) to partition the variance contributed by these uncertainties (Pianosi et al., 2016). As a key mitigation strategy, integrating dynamic land-use models (Meentemeyer et al., 2013) with our projections would identify areas that are both climatically suitable and likely to remain forested. Approximating future soil conditions using pedotransfer functions could further reduce this uncertainty (Smith et al., 2020). This transparent framework aims to advance the predictive accuracy of SDMs for epiphytic species. Orchid seeds are universally minute, contain an undifferentiated embryo, are enclosed by a transparent testa, and lack endosperm; consequently, natural germination is negligible unless colonised by compatible mycorrhizal fungi (Liu et al., 2021; Rasmussen et al., 2015). Field and experimental data confirm that *D. flexicaule* follows this rule, exhibiting almost zero recruitment without fungal partners. Mycorrhizal fungi can either facilitate or constrain plant migration by buffering physiological stress and moderating extinction risk (Bennett and Classen, 2020). Warming and reduced precipitation have been shown to reshape ectomycorrhizal communities (Fernandez et al., 2023) and to enhance the performance of endophytic associations (Wu et al., 2023). Mediterranean work further demonstrates that even minor temperature increases can markedly alter mycorrhizal composition and function (Royal, 2025). These findings underscore that plant microbiomes may mediate, but not guarantee, persistence under climate change (Afkhami et al., 2025). Spatially, *D. flexicaule* habitat consistently broadens in response to warming. An analogous "symbiotic mismatch" could unfold for *D. flexicaule* in the Qinba Mountains: rising temperatures and altered precipitation may reduce the abundance or compatibility of requisite fungal taxa, thereby depressing seedling recruitment despite climatic suitability. This biotic filter represents an ecologically plausible, yet unmeasured, process that could further constrict the species’ realised niche beyond the abiotic projections presented here. Integrating high-resolution data on the distribution and ecological tolerances of key mycorrhizal symbionts is therefore essential to refine conservation strategies for *D. flexicaule* under continued climate change.

## 5 Conclusion

MaxEnt projections under divergent climate scenarios indicate that suitable habitat for the medicinally important orchid *D. flexicaule* will expand markedly across Sichuan, Chongqing, Guizhou, Henan and Hubei, with temperature, slope and precipitation acting as the primary environmental drivers. Overlaying these projections with InVEST-derived habitat quality allowed us to delimit two functional zonings. (1) Priority-conservation area, concentrated in Chengkou County (Chongqing), Fangxian County and the Shennongjia Forest District (Hubei), all located on the southern Daba foothills and eastern Qinling Mountains,should be gazetted as ecological-red-line zones where large-scale infrastructure and mining are banned; eco-compensation schemes and community-based initiatives will reconcile protection with local livelihoods while safeguarding primary habitats. (2) Priority-development areas, centred in Xingshan County (Hubei) and Zhenping County (Shaanxi), should foster community-led, sustainable cultivation coupled with third-party eco-certification and orchid-themed ecotourism to advance rural revitalisation without encroaching on wild populations. An annual GIS-/remote-sensing monitoring platform is recommended to track habitat dynamics in both zone types, enabling adaptive governance of conservation–development trade-offs. This integrated modelling-management framework provides theoretical support for balancing in-situ conservation and regulated utilisation of *D. flexicaule* under future climates.

## Data Availability

The original contributions presented in the study are included in the article/supplementary material. Further inquiries can be directed to the corresponding authors.
